# Supplementary material for: Glutathione determines chronic myeloid leukemia vulnerability to an inhibitor of CMPK and TMPK
Source: Commun Biol. 2024 Jul 10;7:843. doi: 10.1038/s42003-024-06547-1 (PMC11237035; doi:10.1038/s42003-024-06547-1)
Supplement: Supplementary file 2 — Supplementary information [file 42003_2024_6547_MOESM2_ESM.pdf]

Glutathione Determines Chronic Myeloid Leukemia Vulnerability to an Inhibitor of CMPK and TMPK

Chang-Yu Huang[1], Yin-Hsuan Chung[1], Sheng-Yang Wu[1], Hsin-Yang Wang[1], Chih-Yu Lin[2], Tsung-Jung Yang[3], Jim-Ming Fang[3], Chun-Mei Hu[4], and Zee-Fen Chang[1,5*]

[1] Institute of Molecular Medicine, College of Medicine, National Taiwan University, Taipei, 100, Taiwan; [2] Agricultural Biotechnology Research Center, Academia Sinica, Taipei, Taiwan; [3]Institute of Chemistry, National Taiwan University, Taipei, Taiwan; [4]Genomics Research Center, Academia Sinica, Taipei 11529, Taiwan and [5]Center of Precision Medicine, College of Medicine, National Taiwan University

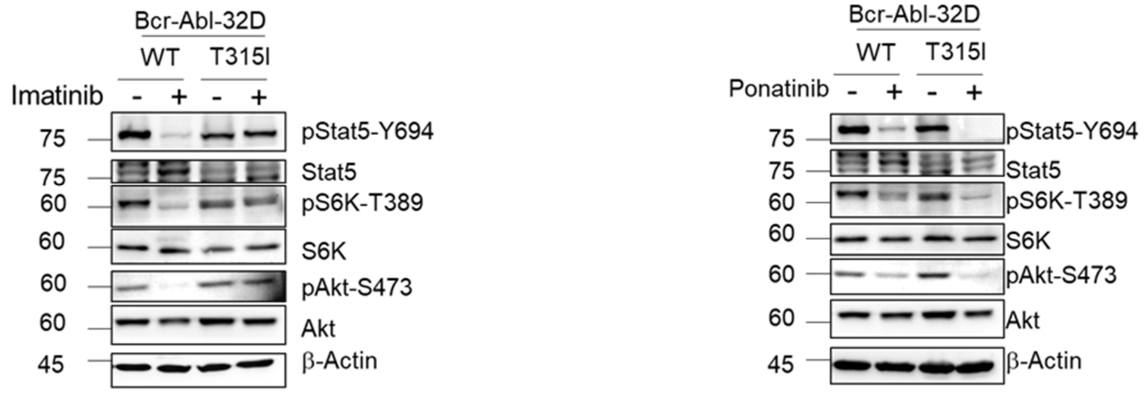

Supplementary Figure 1. Bcr-Abl-activated downstream signaling in WT- and T315I-Bcr-Abl transformed 32D cells.

The comparison of Bcr-Abl downstream signaling (Akt, mTorC1, and Stat5) in WT- and T315I Bcr-Abl-transformed 32D myeloid progenitor cells. After the treatment of imatinib (2 μM) and ponatinib (2 μM) for 4 h, cell lysates were analyzed by Western blot.

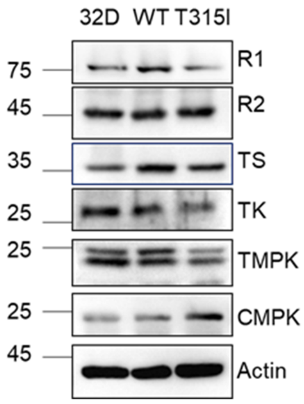

Supplementary Figure 2. Comparison of the expression levels of proteins responsible for dTTP and dCTP synthesis.

Immunoblotting of enzymes for dTTP and dCTP synthesis in untransformed-, WT-, T315I-Bcr-Abl transformed 32D cells.

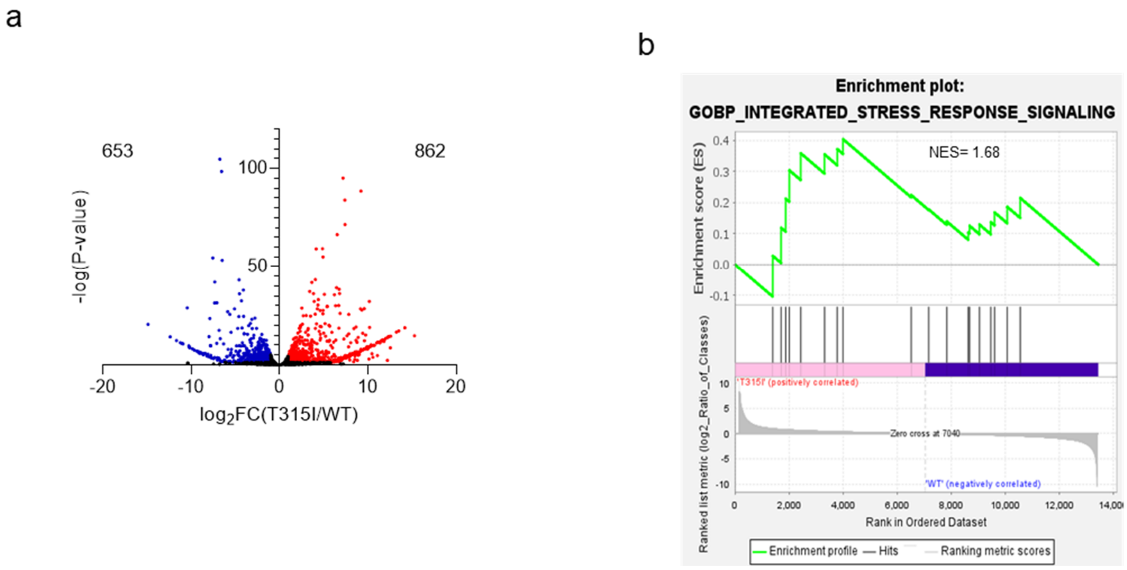

Supplementary Figure 3. RNA-seq analysis of WT- and T315I-Bcr-Abl-32D cells. **a)** RNA was extracted from WT- and T315I-Bcr-Abl-32D cells for RNA-seq analysis. The volcano plot illustrates the comparison between T315I and WT Bcr-Abl-32D cells. Significantly upregulated genes (log2(fold change) ≥ 1; $p < 0.05$) in T315I-Bcr-Abl-32D cells are labeled in red, while significantly downregulated genes (log2(fold change) ≥ 1; $p < 0.05$) are represented in blue. **b)** Gene set enrichment analysis (GSEA) of Gene Ontology (GO) pathways using RNA-sequencing data of WT- and T315I-Bcr-Abl-32D cells. An "Integrated stress response" enrichment in T315I-Bcr-Abl-32D cells. The plot depicts the running Normalized Enrichment Score (NES) and gene set members' positions in the rank-ordered list.

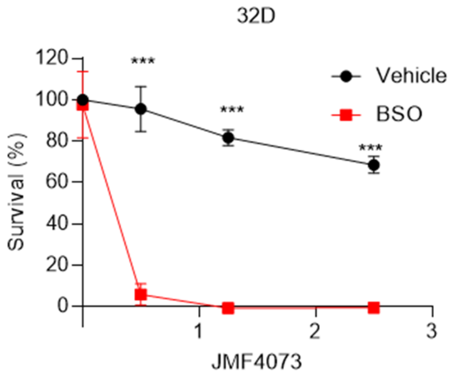

Supplementary Figure 4. BSO treatment synergistically inhibits the growth of untransformed 32D cells in combination with JMF4073.

32D cells were pre-treated with BSO (12.5 μM) for 16 h prior to JMF4073 incubation. After 72 h, viability assays were performed. Data are represented as means ± S.D. from 3 independent experiments. Asterisks denote ***p<0.001 from non-paired two-tailed Student's t test.

Supplemental Table 1. Hematology analysis of leukemia-bearing (WT-Bcr-Abl-32D) mice with or without JMF4073 treatment by submandibular blood collection.

Day 26	WBC	RBC	HGB	RET	PLT
Normal mice	4.12	9.63	15.5	511.4	558
IV: Bcr-Abl-32D<br>IP: Vehicle	WBC	RBC	HGB	RET	PLT
1	46.82	5.51	9.8	1959.4	107
2	15.53	7.27	11.8	594.7	37
3	20.88	5.67	9.6	1561	62
4	12.62	6.01	9.8	540.9	8
5	6.08	7.21	11.9	792.4	546
6	7.69	6.85	10.9	437.7	43
7	3.34	6.89	12.5	1111.4	505
8	3.9	7.5	12.8	804	464
IV: Bcr-Abl-32D<br>IP: JMF 4073	WBC	RBC	HGB	RET	PLT
1	2.76	6.89	12	798.6	381
2	117.06	3.85	6.5	2539.1	16
3	4.04	7.43	12.1	630.1	666
4	6	7.9	12.9	708.6	602
5	3.99	5.96	9.4	501.2	10
6	38.58	6.15	10.4	2043	161

WBC (White blood cell/Leukocyte count); RBC (Red blood cell/ Erythrocyte count), HGB (Hemoglobin Concentration); RET (Reticulocytes count); PLT (Platelet/ Thrombocyte).

Supplemental Table 2. Hematology analysis of leukemia-bearing (T315I-Bcr-Abl-32D) mice with or without JMF4073 treatment by submandibular blood collection.

IV: T315I-Bcr-Abl-32D IP: Vehicle	WBC	RBC	HGB	RET	PLT
1	6.31	8.37	13.3	733.2	739
2	6.11	8.48	13.4	749.6	745
3	6	8.34	13.4	528.8	607
4	2.92	6.29	10.9	719.6	384
5	6.19	7.71	12.3	518.1	751
6	6.9	8.22	13.1	655.1	661
7	4.51	5.39	8.7	2006.2	10
**IV: T315I-Bcr-Abl-32D IP: JMF 4073**	**WBC**	**RBC**	**HGB**	**RET**	**PLT**
1	1.8	7.01	11.8	461.3	369
2	4.88	8.15	12.8	502.9	657
3	5.4	8.64	13.7	530.5	708
4	3.86	7.25	12.4	634.4	371

WBC (White blood cell/Leukocyte count); RBC (Red blood cell/ Erythrocyte count), HGB (Hemoglobin Concentration); RET (Reticulocytes count); PLT (Platelet/ Thrombocyte).

Supplementary methods

Oligonucleotides used for dNTP quantitation

RCA-qPCR assay for dNTP pool measurement		
Probe	Sequence 5'-3'	Modification
Primer	CTCTG GTGTG AGTCC CAAGT CCTGG ACTAT	None
Padlock probe	GGACT CACAC CAGAG GATGA TTCTG TGATC CAACG TCTTC G AAAAA ATAGT CCAGG ACTTG	5'-phosphate
qPCR-F	CTCTG GTGTG AGTCC CAAG	None
qPCR-R	CACAC CAGAG GATGA TTCTG	None

Primers used in RT-qPCR for RNA sequencing validation

RT-qPCR		
Gene symbol	Forward primer	Reverse primer
Mgst2	GAAAGATGGCCGGGGATTCAAG	ACCCAGACAGGCTGCAAAAAC
Mgst3	TTTACCACCCGCGCATAGCTTC	ATGCTGGAAAGCAGAGCACAC
Gsta4	AGCTCAGTTGGGCAGACATC	AGCACGCTGCACTAGAACTT
GAPDH	TGCCCCCATGTT TGTGATG	TGTGGTCATGAGCCCTTCC
Slc1a1	CCTCATCTCCAGCCACTCTCAAAC TACC	AAACCCTTTCCCTTCCCCTAAACC CC
Slc7a8	AAGAAGCCTGACATTCCCCG	TGTGTTGCCAGTAGACACCC
Psat1	TCCAGAAGCAGCTACTAGACTACA GAGGAC	GCCAATCAGATTTAAGGGGACAGC ACTGAAC
Shmt2	TGGAACTCGTCTCCATCACAGC	GACTCTACGGAAGTCGTCCTCA

Materials

Antibodies: Specific primary antibodies: Akt (2532S, 1:2000), pS473-Akt (9271S, 1:1000), S6K (9202S, 1:2000), pT389-S6K (9205S, 1:1000), and Stat5 (9420S, 1:10000), pY694-Stat5 (9356S, 1:1000 were obtained from Cell Signaling. Anti-phospho-Tyr99 (sc-7020, 1:1000) from Santa Cruz.; β-Actin was from GeneTex (GTX109639, 1:1000); RRM1 (sc-11733, 1:1000), and RRM2 (sc-10846, 1:1000) from Santa Cruz. anti-TMPK and anti-CMPK were prepared as described previously(1).

Mouse CML induction and in vivo therapy

The animal studies were approved by the biosafety committee at National Taiwan University and conformed to the national guidelines and regulations (IACUC # 20201063). Female C3H/HeNCrNarl mice were used at 6-8 weeks of age (National Laboratory Animal Center, Taiwan). WT-Bcr-Abl-32D-EGFP+ cells (5×10^5) suspended in 200 μL of HBSS were injected into each mouse through the tail vein. After 48 h of transplantation, mice were treated with vehicle (DMSO), or JMF4073 (5 mg/kg body weight) by intraperitoneal injection at 24 h interval for 14 days. Injected mice were monitored for peripheral blood (PB) and counted by Taiwan Mouse Clinic-National Phenotyping Center, National Research Program for Genomic Medicine (NSC). For T315I-Bcr-Abl CML mice, T315I-Bcr-Abl-32D-EGFP+ cells (1×10^6) suspended in 200 μL of HBSS were tail-vein-injected. After 7 days of transplantation, mice were treated with vehicle (DMSO), UK-5099 (10 mg/kg body weight), JMF4073 (5 mg/kg body weight), or UK-5099 combined with JMF4073 by intraperitoneal injection at 24 h interval for 14 days. For monitoring CML progress, mice blood was collected in anticoagulation tubes by submandibular blood collection, followed by lysing red blood cells with RBC lysis buffer (Invitrogen). After washing, the remaining cells were suspended in 500 μL of HBSS and subjected to flow cytometry analysis (FACScalibur, BD Bioscience) with CellQuest software. The number of EGFP+ cells

were collected from 20,000 single-cell events.

Metabolic flux by U-^{13}C-glutamine tracing measurement

Cells in 10-cm dishes were washed twice for metabolite tracing by glutamine-free RPMI-1640 medium. For glutamine tracing, 4 mM of U-^{13}C-glutamine was added to the glutamine-free RPMI-1640 medium with 10% v/v HI-FBS, 1 mM HEPES, and 1 mM sodium pyruvate. After 2 h incubation, cells were washed twice with cold PBS and were extracted with 80% ice-cold methanol at 2x10^{6} cells/mL. Samples were incubated at -80℃ for overnight. After centrifugation, supernatants were transferred to fresh tubes and evaporated using a speed-vac.

Agilent 1290 Infinity II ultra-performance liquid chromatography (UPLC) system (Agilent Technologies, Palo Alto, CA, USA) coupled online to the Dual AJS electrospray ionization (ESI) source of an Agilent 6545XT quadrupole time-of-flight (Q-TOF) mass spectrometer (Agilent Technologies, Palo Alto, CA, USA) was used for the analysis. The ACQUITY UPLC BEH amide column (1.7 μm, 2.1 × 100 mm, Waters Corp., Milford, MA, USA) at 40℃ was employed for sample separation. The mobile phase was composed of double-distilled water (eluent A) and 10% double-distilled water in acetonitrile (eluent B) eluted with 15 mM ammonium acetate and 0.3% $NH_3{\cdot}H_2O$. The flow rate was 300 μL/min, and the sample injection volume was 2 μL. The instrument was operated in positive and negative full-scan mode, collected from a m/z of 60 – 1500. The MS operating conditions were optimized as follows: Vcap voltage, 3.5 kV; nozzle voltage, 1 kV for negative mode and 0 V for positive mode; nebulizer, 45 psi; gas temperature, 200℃; sheath gas temperature, 300℃; sheath gas flow (nitrogen), 8 L/min; drying gas (nitrogen), 10 L/min. The chromatogram acquisition, mass spectral peaks detection, and waveform processing were performed using Agilent Qualitative Analysis 10.0 and Agilent Profinder 10.0 software (Agilent, USA).

Measurement of dNTP pools by RCA-qPCR assay

Cells (10^6) were extracted with 1 ml of ice-cold 80% methanol at -80 ℃ for 16 hr, followed by heating at 95℃ for 3 minutes. The methanol extracts were centrifuged (16,000 x g, 30 min) to remove cell debris. For chloroform extraction, same volume of chloroform was added to the methanol extract and vortexed. After phase separation, upper phase was collected and vacuum-dried. The dried residues were dissolved in water, in which 1x104 cells/ 5μL were used for RCA-qPCR assays as described previously. Briefly, cell extracts were added to the RCA reaction mixture (10 μL) containing 50 mM Tris-HCl, 10 mM $MgCl_2$, 10 mM $(NH_4)_2SO_4$, 4 mM DTT, 0.1% Tween-20, pH 7.4, 2 units of phi29 DNA polymerase (New England Biolabs) in the presence of three not to quantified deoxynucleoside triphosphate mix (20 μM), and 1 attomol of the annealed template-primer. The reaction was incubated at 37˚C for 1 h and terminated by heating at 65˚C for 10 min. The RCA products were determined by the subsequent addition of another 15 μL of qPCR reaction mixture (PCR Biosystem). Oligonucleotides used in RCA-qPCR are listed in Supplementary methods.

Synthesis of JMF4073

As shown in Scheme 1, we first carried out an S_NAr reaction of 2,5-difluorobenzonitrile with sodium sulfide to obtain a mixture of the thiol compound **3** and the disulfide **3**-dimer. Thus, Zn powder in 1 M $HCl_{(aq)}$ was used to reduce the disulfide bond. The overall yield of the desired thiophenol **3** was 81%. The thiophenol compound **3** was then heated in concentrated H_2SO_4 to furnish an oxidative cyclization product **4** (84% yield). The substitution reaction of *tert*-butyl 4-(2-chloroacetyl)piperazine-1-carboxylate (compound **5**) with the isothiazolone compound **4** was realized by the promotion of sodium iodide to afford the desired product **1** (JMF4073) in high purity (98.5%) after silica gel chromatography.

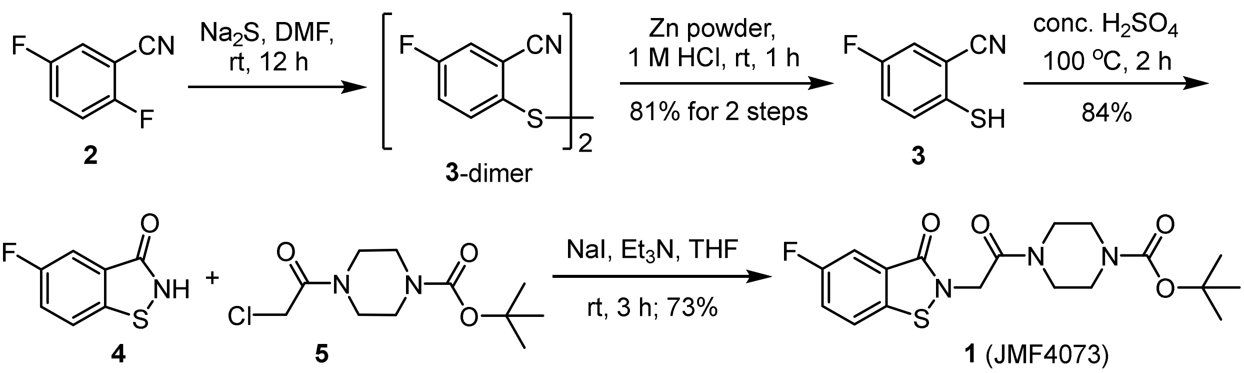

Scheme 1. Synthesis of isothiazolone JMF4073

5-Fluoro-2-mercaptobenzonitrile (3)

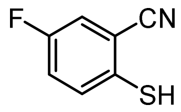

A mixture of 2,5-difluorobenzonitrile (**2**) (139 mg, 1.0 mmol) and Na_2S (94 mg, 1.2 mmol) in DMF (3 mL) was stirred at room temperature for 16 h. The mixture was extracted with Et_2O and 1 M $HCl_{(aq)}$. The organic phase was washed with brine, dried over $MgSO_4$, filtered, and concentrated under reduced pressure to give a mixture of the thiol compound **3** and the disulfide **3**-dimer. To the mixture in an ice-bath were added 1 M $HCl_{(aq)}$ (3 mL) and EtOAc (5 mL). Then, zinc powder (100 mg) was slowly added, and the mixture was stirred for another 1 h at room temperature. The aqueous and organic layers were separated. The aqueous layer was acidified to pH 1–2 with 3 M $HCl_{(aq)}$, and then extracted with CH_2Cl_2. The organic layer was treated with 1 M $NaOH_{(aq)}$, and then extracted with CH_2Cl_2. The combined organic phase was dried over $MgSO_4$, filtered, and concentrated under reduced pressure to give the thiol compound **3** (124 mg, 81% yield). C_7H_4FNS; white solid; mp 83–84 °C; TLC (hexane/EtOAc = 1:1) R_f = 0.15; IR ν_{max} (KBr) 2919, 2854, 2564, 2229, 1468, 1400, 1233, 1146 cm^{-1}; ^1H NMR (400 MHz, $CDCl_3$) δ 7.40–7.37 (1 H, m), 7.31–7.29 (1 H, m), 7.20–7.15 (1 H, m), 4.00 (1 H, s); ^{13}C NMR (100 MHz, $CDCl_3$) δ 160.1 (d, J_{F-C} = 249 Hz), 132.3 (d,

J_{F-C} = 3.2 Hz), 131.7 (d, J_{F-C} = 7.9 Hz), 121.0 (d, J_{F-C} = 22.0 Hz), 120.1 (d, J_{F-C} = 25.1 Hz), 116.3 (d, J_{F-C} = 2.7 Hz), 113.5 (d, J_{F-C} = 9.2 Hz); ^{19}F (374 MHz, $CDCl_3$) δ –115.3.

5-Fluorobenzo[*d*]isothiazol-3(2*H*)-one (4)

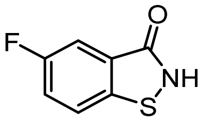

A mixture of the thiophenol compound **3** (153 mg, 1.0 mmol) and conc. H_2SO_4 (2 mL) was immersed in a preheated oil bath at 100 °C for 2 h. The mixture was cooled, and modulated to pH 5–6 by addition of 2 M $NaOH_{(aq)}$ to produce insoluble substance in suspension. The solids were collected by filtration, and dried under reduced pressure to give the desired product **4** (142 mg, 84% yield). C_7H_4FNOS; yellowish solid; mp 184–185 °C; TLC (hexane/EtOAc = 1:1) R_f = 0.48; IR ν_{max} (KBr) 2916, 1639, 1464, 1332, 1272, 1105 cm^{-1}; ^{1}H NMR (400 MHz, DMSO-d_6) δ 11.82 (1 H, br s), 8.06–8.03 (1 H, m), 7.64–7.61 (1 H, m), 7.56–7.51 (1 H, m); ^{13}C NMR (100 MHz, DMSO-d_6) δ 163.8 (d, J_{F-C} = 3.4 Hz), 160.2 (d, J_{F-C} = 241.8 Hz), 143.8, 125.9 (d, J_{F-C} = 8.6 Hz), 123.5 (d, J_{F-C} = 8.9 Hz), 118.8 (d, J_{F-C} = 25.2 Hz), 109.2 (d, J_{F-C} = 23.2 Hz); ^{19}F NMR (374 MHz, DMSO-d_6) δ –119.0; ESI–HRMS calcd for C_7H_5FNOS: 170.0062, found: *m/z* 170.0070 $[M + H]^+$.

***Tert*-butyl 4-(2-(5-fluoro-3-oxobenzo[*d*]isothiazol-2(3*H*)-yl)acetyl)piperazine-1-carboxylate (compound 1, JMF4073)**

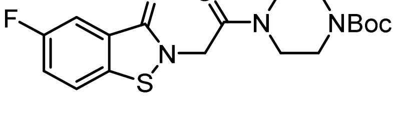

A mixture of the isothiazolone compound **4** (17 mg, 0.10 mmol), *tert*-butyl 4-(2-chloroacetyl) piperazine-1-carboxylate(2)(compound **5**) (26 mg, 0.10 mmol), NaI (90 mg, 0.60 mmol) and Et_3N (30 μL, 0.22 mmol) in THF (1 mL) was stirred for 3 h at

room temperature. The mixture was concentrated under reduced pressure, and the residue was extracted with CH_2Cl_2 and H_2O. The organic phase was dried over $MgSO_4$, filtered, concentrated under reduced pressure, and purified by silica gel chromatography (hexane/EtOAc = 1:1) to give compound **1** (JMF4073) (29 mg, 73% yield). The purity of compound **1** was 98.5% as shown by HPLC on an HC-C18 column (Agilent, 4.6 × 250 mm, 5 μm particle size), t_R = 4.3 min by elution with hexane/EtOAc = 4:6 at a flow rate of 1.0 mL/min. $C_{18}H_{22}FN_3O_4S$; white solid; mp 226–227 °C; TLC (hexane/EtOAc = 1:1) R_f = 0.23; IR ν_{max} (KBr) 2963, 2866, 1692, 1657, 1466, 1368, 1237, 1169, 1035 cm^{-1}; ^{1}H NMR (400 MHz, $CDCl_3$) δ 7.69 (1 H, dd, J = 7.9, 2.2 Hz), 7.52–7.48 (1 H, m), 7.36 (1 H, td, J = 8.6, 2.6 Hz), 3.60–3.58 (2 H, m), 4.68 (2 H, s), 3.53–3.51 (2 H, m), 3.46–3.40 (4 H, m), 1.44 (9 H, s); ^{13}C NMR (100 MHz, $CDCl_3$) δ 164.9 (2 ×), 161.0 (d, J_{F-C} = 246.4 Hz), 154.3, 136.5, 124.7 (d, J_{F-C} = 8.5 Hz), 121.9 (d, J_{F-C} = 8.1 Hz), 121.0 (d, J_{F-C} = 25.0 Hz), 112.6 (d, J_{F-C} = 23.7 Hz), 80.4, 45.0, 44.9 (2 ×), 41.9 (2 ×), 28.3 (3 ×); ^{19}F NMR (374 MHz, DMSO-d_6) δ –116.9; ESI–HRMS calcd for $C_{18}H_{23}FN_3O_4S$: 396.1393, found: m/z 396.1375 $[M + H]^+$.

NMR spectra and HPLC diagram

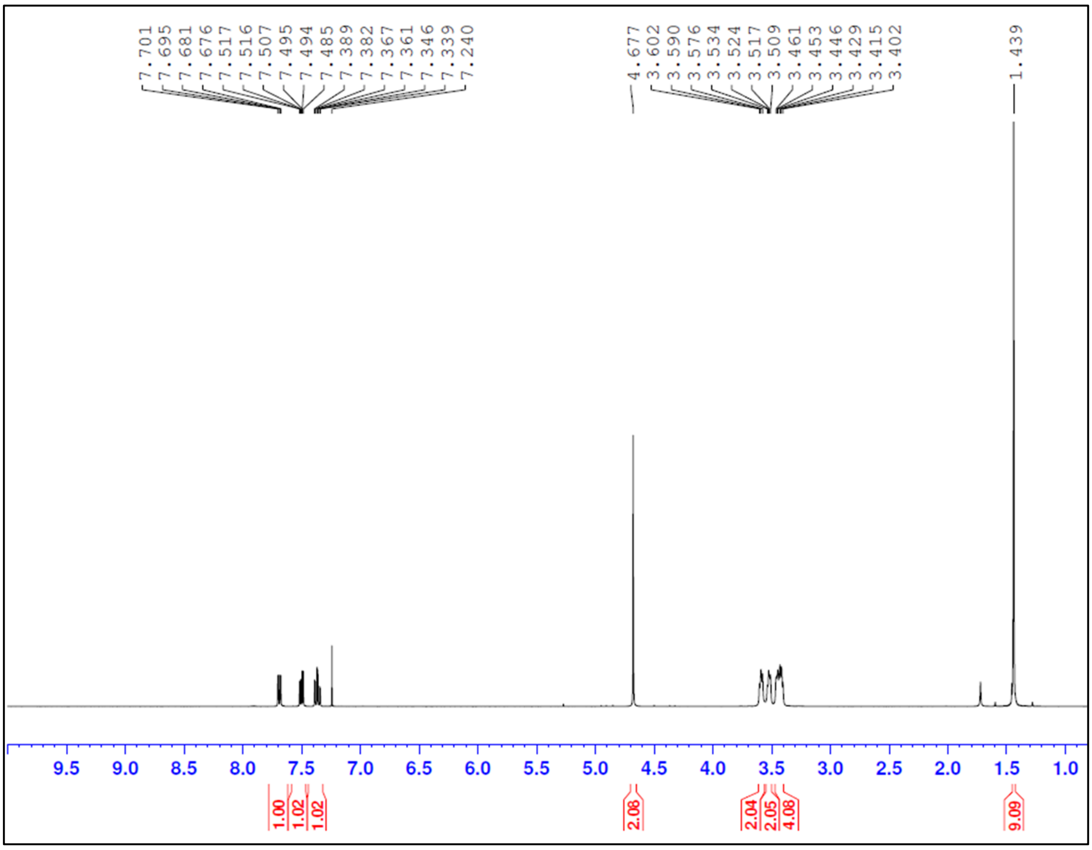

^1H NMR spectrum (400 MHz, $CDCl_3$) of JMF4073

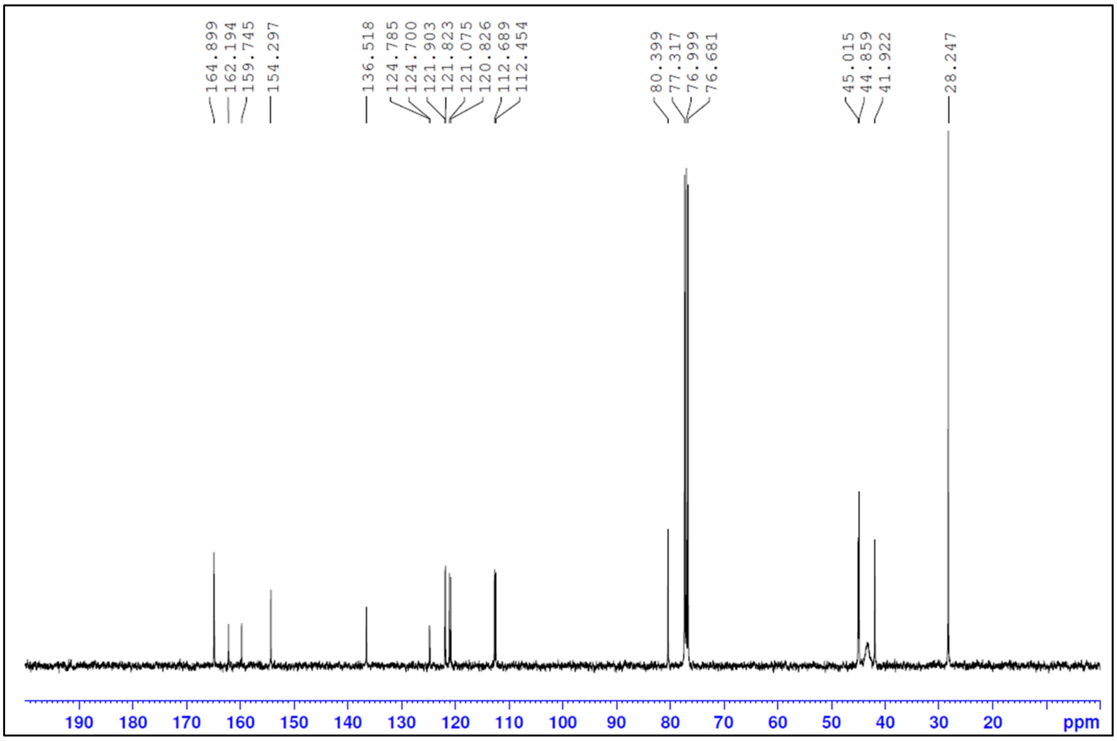

^{13}C NMR spectrum (100 MHz, $CDCl_3$) of JMF4073

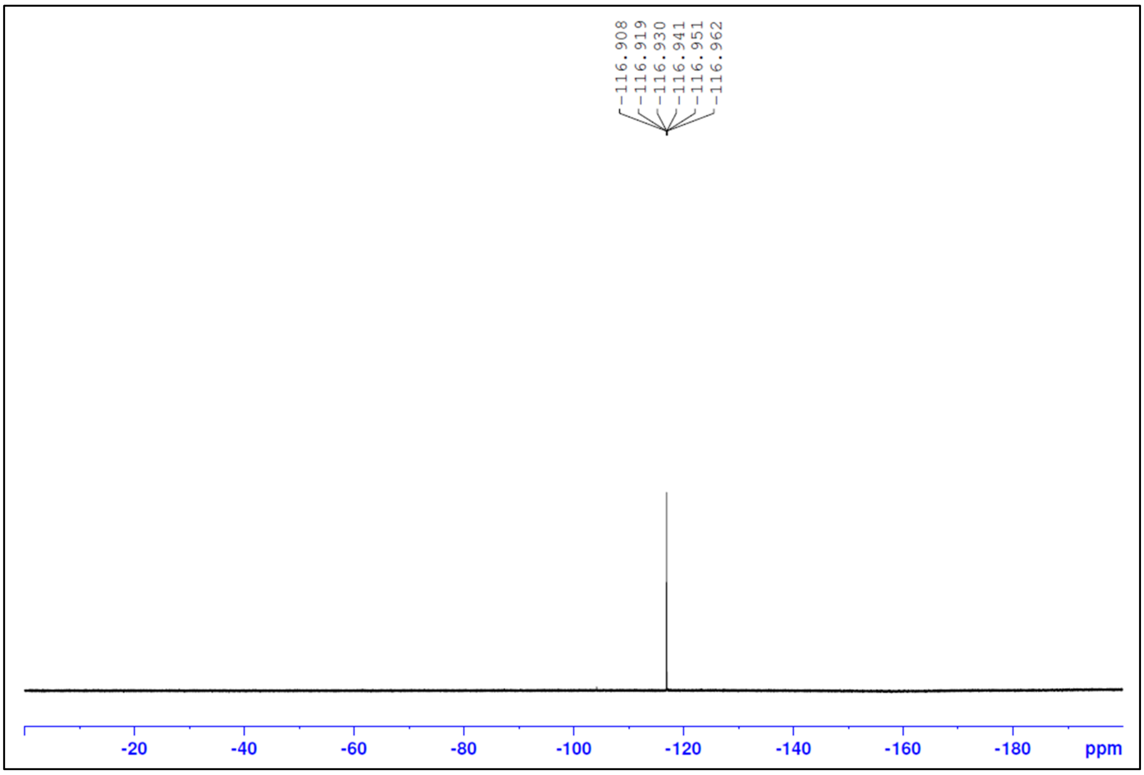

^{19}F NMR spectrum (374 MHz, $CDCl_3$) of JMF4073

```
=====================================================================
Injection Date  : 6/2/2020 3:16:27 PM
Sample Name     :                                 Location : Vial 1
Acq. Operator   : JMF
Acq. Instrument : Instrument 1
Acq. Method     : C:\HPCHEM\1\METHODS\DEF LC.M
Last changed    : 6/2/2020 3:16:04 PM by JMF
                  (modified after loading)
Analysis Method : C:\HPCHEM\1\METHODS\DEF LC.M
```

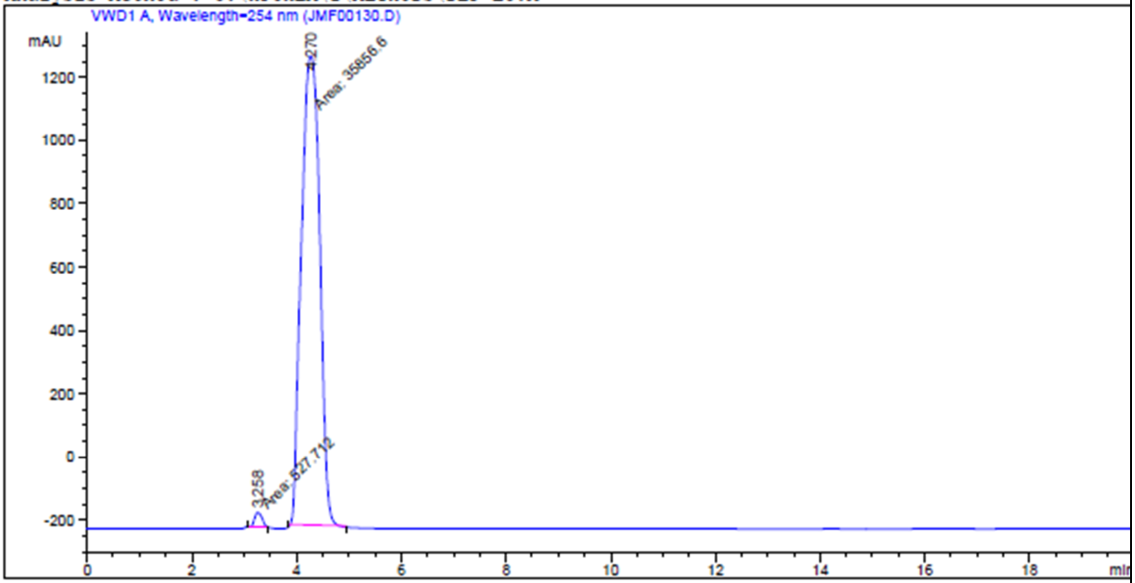


```
=====================================================================
                         Area Percent Report
=====================================================================

Sorted By             :      Signal
Multiplier            :      1.0000
Dilution              :      1.0000
Use Multiplier & Dilution Factor with ISTDs

Signal 1: VWD1 A, Wavelength=254 nm

Peak RetTime Type  Width     Area      Height     Area
  #   [min]        [min]  mAU    *s   [mAU   ]      %
----|-------|----|-------|----------|----------|--------|
   1   3.258 MM    0.1795  527.71155   48.99210   1.4504
   2   4.270 MM    0.4033 3.58566e4  1481.91663  98.5496

Totals :                  3.63843e4  1530.90872

 Results obtained with enhanced integrator!
=====================================================================
                         *** End of Report ***
```

HPLC diagram of JMF4073. HC-C18 column (Agilent, 4.6 × 250 mm, 5 μm particle size), Hexane/EtOAc = 4:6 elution at a flow rate of 1.0 mL/min, t_R = 4.3 min, purity 98.5%.

Reference

1. Ke PY, Kuo YY, Hu CM, Chang ZF. Control of dTTP pool size by anaphase promoting complex/cyclosome is essential for the maintenance of genetic stability. Genes Dev. 2005;19(16):1920-33.

2. Chen YH, Hsu HY, Yeh MT, Chen CC, Huang CY, Chung YH, et al. Chemical Inhibition of Human Thymidylate Kinase and Structural Insights into the Phosphate Binding Loop and Ligand-Induced Degradation. J Med Chem. 2016;59(21):9906-18.

Western data

Fig 2a

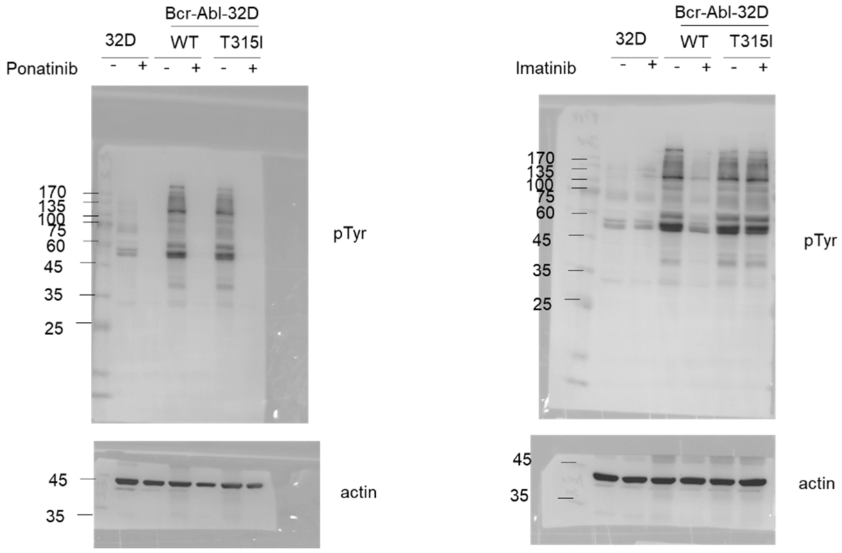

Fig 2c

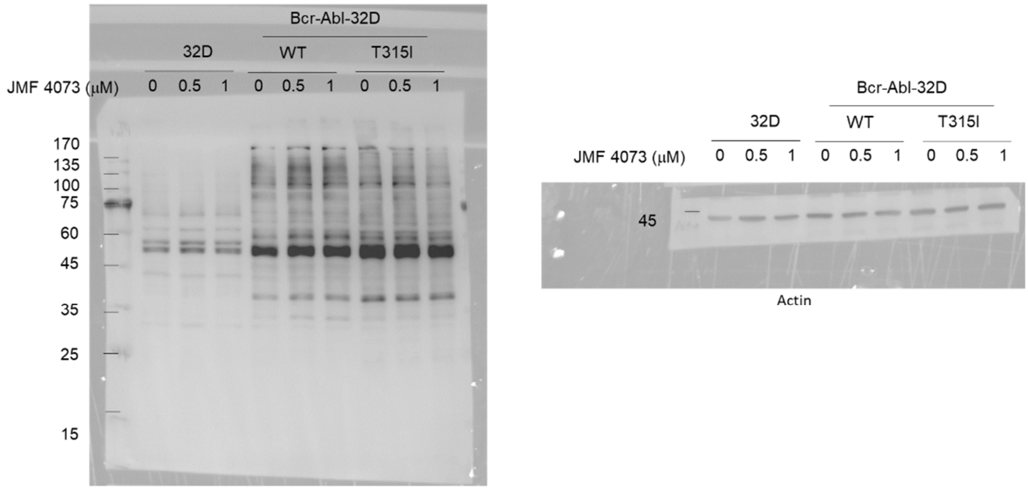

Fig 4e

Fig 4d

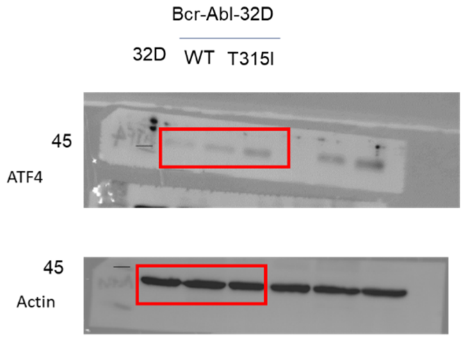

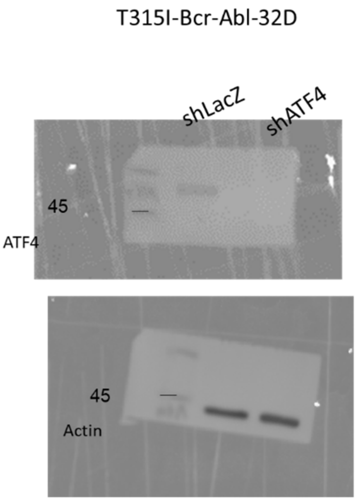

Fig 7b

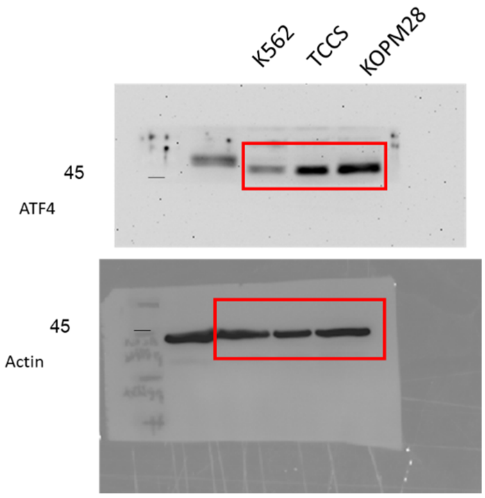

Fig S1

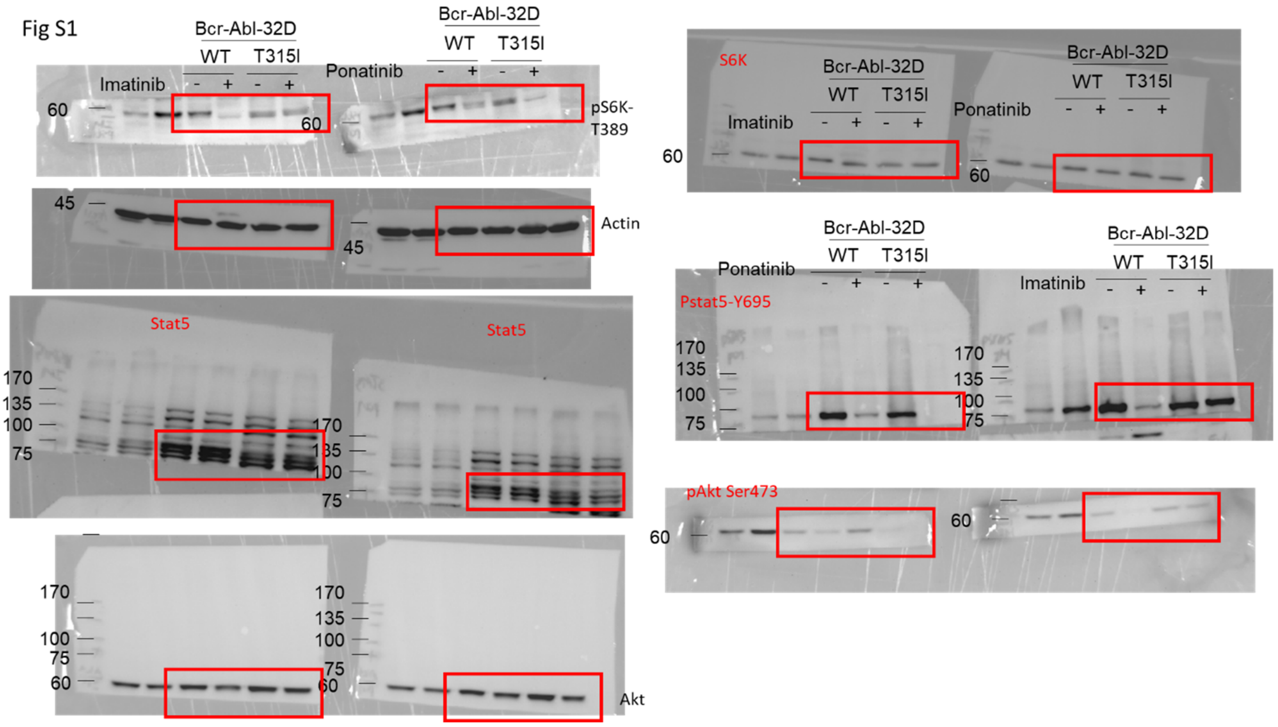
Bcr-Abl-32D
WT T315I
Imatinib - + - +
Ponatinib - + - +
pS6K-T389
Actin
Stat5
Stat5
Akt
S6K
Pstat5-Y695
pAkt Ser473
170
135
100
75
60
45

Fig S2

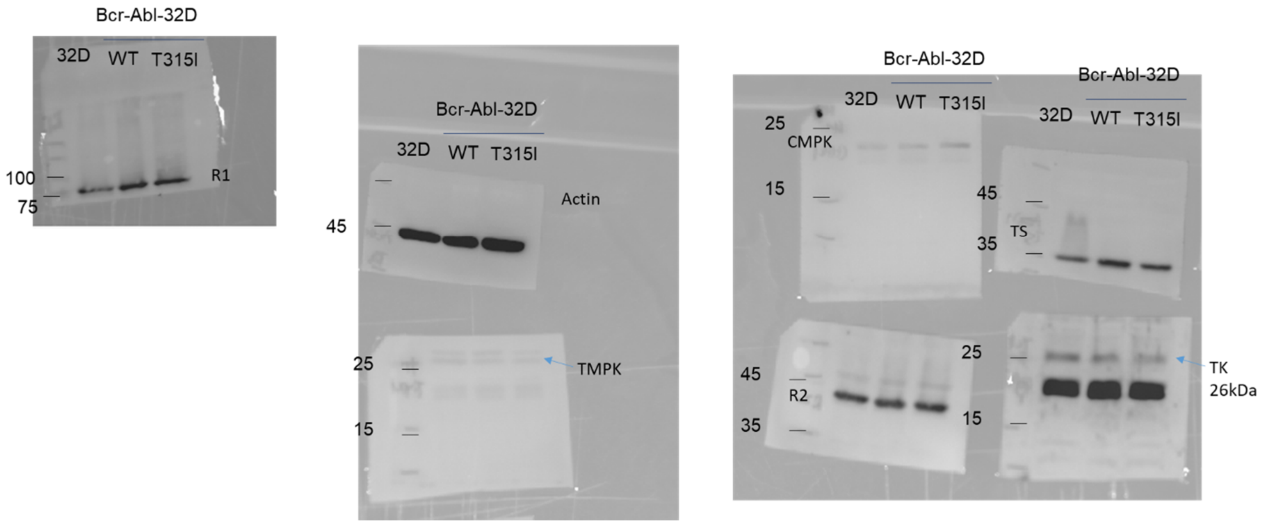
Bcr-Abl-32D
32D WT T315I
R1
Actin
TMPK
CMPK
TS
R2
TK
26kDa
100
75
45
25
15
35

Gating Strategy in Figure 3c,

We utilized unstained cells as the control for CellRox staining. Mean intensity was calculated using CellQuest Pro.

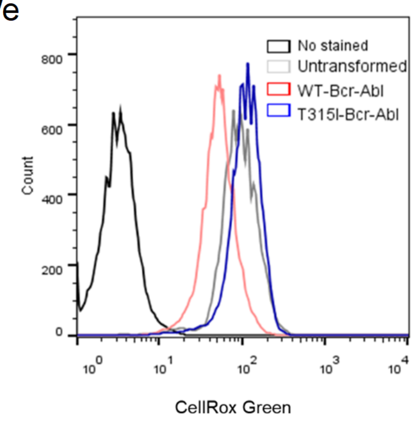

Gating Strategy in Figure 6g

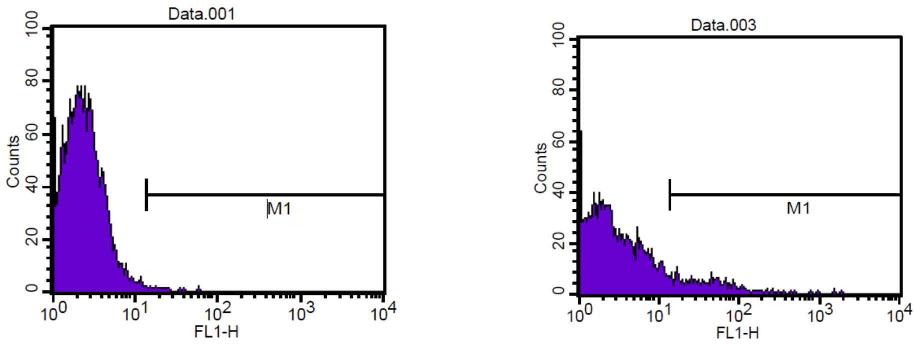

Left: Peripheral blood cells from mice without induced CML.
Right: Peripheral blood cells from mice induced with CML (via tail vein injection with T315I-Bcr-Abl-32D/EGFP cells).
Calculate the number of EGFP+ cells in M1 gating using CellQuest Pro.
