# Supplementary material for: Glutathione determines chronic myeloid leukemia vulnerability to an inhibitor of CMPK and TMPK
Source: Commun Biol. 2024 Jul 10;7:843. doi: 10.1038/s42003-024-06547-1 (PMC11237035; doi:10.1038/s42003-024-06547-1)
Supplement: Supplementary file 5 — Reporting Summary [file 42003_2024_6547_MOESM5_ESM.pdf]

nature portfolio

Corresponding author(s): Zee-Fen Chang

Last updated by author(s): 2024/01/31

Reporting Summary

Nature Portfolio wishes to improve the reproducibility of the work that we publish. This form provides structure for consistency and transparency in reporting. For further information on Nature Portfolio policies, see our Editorial Policies and the Editorial Policy Checklist.

Statistics

For all statistical analyses, confirm that the following items are present in the figure legend, table legend, main text, or Methods section.

n/a	Confirmed	
☐	☒	The exact sample size (*n*) for each experimental group/condition, given as a discrete number and unit of measurement
☐	☒	A statement on whether measurements were taken from distinct samples or whether the same sample was measured repeatedly
☐	☒	The statistical test(s) used AND whether they are one- or two-sided<br>*Only common tests should be described solely by name; describe more complex techniques in the Methods section.*
☒	☐	A description of all covariates tested
☐	☒	A description of any assumptions or corrections, such as tests of normality and adjustment for multiple comparisons
☐	☒	A full description of the statistical parameters including central tendency (e.g. means) or other basic estimates (e.g. regression coefficient) AND variation (e.g. standard deviation) or associated estimates of uncertainty (e.g. confidence intervals)
☒	☐	For null hypothesis testing, the test statistic (e.g. *F*, *t*, *r*) with confidence intervals, effect sizes, degrees of freedom and *P* value noted<br>*Give P values as exact values whenever suitable.*
☒	☐	For Bayesian analysis, information on the choice of priors and Markov chain Monte Carlo settings
☒	☐	For hierarchical and complex designs, identification of the appropriate level for tests and full reporting of outcomes
☐	☒	Estimates of effect sizes (e.g. Cohen's *d*, Pearson's *r*), indicating how they were calculated

Our web collection on statistics for biologists contains articles on many of the points above.

Software and code

Policy information about availability of computer code

Data collection	NA
Data analysis	NA

For manuscripts utilizing custom algorithms or software that are central to the research but not yet described in published literature, software must be made available to editors and reviewers. We strongly encourage code deposition in a community repository (e.g. GitHub). See the Nature Portfolio guidelines for submitting code & software for further information.

Data

Policy information about availability of data

All manuscripts must include a data availability statement. This statement should provide the following information, where applicable:

- Accession codes, unique identifiers, or web links for publicly available datasets
- A description of any restrictions on data availability
- For clinical datasets or third party data, please ensure that the statement adheres to our policy

All data associated with this study are present in this paper or the supplementary information. RNA sequencing data are deposited in the National Center for Biotechnology Information’s Sequence Read Archive (PRJNA812502) (https://www.ncbi.nlm.nih.gov/sra/?term=PRJNA812502).

Research involving human participants, their data, or biological material

Policy information about studies with human participants or human data. See also policy information about sex, gender (identity/presentation), and sexual orientation and race, ethnicity and racism.

Reporting on sex and gender	NA
Reporting on race, ethnicity, or other socially relevant groupings	NA
Population characteristics	NA
Recruitment	NA
Ethics oversight	NA

Note that full information on the approval of the study protocol must also be provided in the manuscript.

Field-specific reporting

Please select the one below that is the best fit for your research. If you are not sure, read the appropriate sections before making your selection.

☒ Life sciences ☐ Behavioural & social sciences ☐ Ecological, evolutionary & environmental sciences

For a reference copy of the document with all sections, see nature.com/documents/nr-reporting-summary-flat.pdf

Life sciences study design

All studies must disclose on these points even when the disclosure is negative.

Sample size	Sample size choice was based on previous studies, not predetermined by a statistical method.
Data exclusions	No data was excluded.
Replication	Statistical analysis was carried out using Prism 8 (GraphPad Software). Statistical analysis was conducted on data from three or more biologically independent experimental replicates. Comparisons between groups were planned before statistical testing and target effect sizes were not predetermined. Error bars displayed on graphs represent the mean ± SD of at least three independent experiments. Statistical significance was analyzed using unpaired student's t test between two different groups or Mann-Whitney test when samples sizes were small. All tests were two sided.*p<0.05, **p<0.01, and ***p<0.001 were considered significant
Randomization	No randomization method was used.
Blinding	Data collection and Analysis was not performed blind.

Reporting for specific materials, systems and methods

We require information from authors about some types of materials, experimental systems and methods used in many studies. Here, indicate whether each material, system or method listed is relevant to your study. If you are not sure if a list item applies to your research, read the appropriate section before selecting a response.

Materials & experimental systems

n/a	Involved in the study	
☐	☒	Antibodies
☐	☒	Eukaryotic cell lines
☒	☐	Palaeontology and archaeology
☐	☒	Animals and other organisms
☒	☐	Clinical data
☒	☐	Dual use research of concern
☒	☐	Plants

Methods

n/a	Involved in the study	
☒	☐	ChIP-seq
☐	☒	Flow cytometry
☒	☐	MRI-based neuroimaging

Antibodies

Antibodies used

Akt (9272S, 1:2000), pS473-Akt (9271S,1:1000), S6K (9202S, 1:2000), pT389-S6K (9205S, 1:1000), Stat5 (9420S, 1:10000),and pY694-Stat5 (9356S, 1:1000) were obtained from Cell Signaling. Anti-phospho-Tyr99 (sc- 7020, 1:1000) from Santa Cruz. beta-Actin GeneTex (GTX109639, 1:1000); RRM1 (sc-11733, 1:1000), and RRM2 (sc-10846, 1:1000) from Santa Cruz. anti-TMPK and anti-361 CMPK were prepared as described previously (ref 29).MPC1 (14462S, 1:1000) and MPC2 (46141S, 1:1000) were from cell signaling.

Validation

Akt (RRID:AB_329827), pS473-Akt (RRID:AB_329825), S6K (RRID:AB_33167), pT389-S6K (RRID:AB_330944), pY694-Stat5 (RRID:AB_331263). Anti-phospho-Tyr99 (RRID:AB_628123) ,beta-Actin (RRID:AB_1949572); RRM1 (RRID:AB_2180388), RRM2 (RRID:AB_670989), Data providedMPC1 (RRID:AB_2773729), and MPC2 (RRID:AB_2799295) .
Data provided in manuscript.

Eukaryotic cell lines

Policy information about cell lines and Sex and Gender in Research

Cell line source(s)

Murine WEHI, 32D (32Dcl3), WT, and T315I-BcrAbl-transformed 32D cells were generously provided by Dr. Robert Arlinghaus (The University of Texas MD Anderson Cancer Center, Houston, TX, USA.). K562 cells were obtained from BCRC (Bioresource Collection and Research Center, Taiwan), and TCCS/KOPM28 from Takeshi Inukai (Department of Pediatrics, School of Medicine, University of Yamanashi, Chuo, Japan). HEK-293T were from American Type Culture Collection (ATCC)

Authentication

All cell lines were not authentication.

Mycoplasma contamination

All cell lines tested were negative for Mycoplasma contamination.

Commonly misidentified lines
(See ICLAC register)

No.

Animals and other research organisms

Policy information about studies involving animals; ARRIVE guidelines recommended for reporting animal research, and Sex and Gender in Research

Laboratory animals

C3H/HeNCrNarl mice were used at 6-8 weeks of age (National Laboratory Animal Center, Taiwan).WT-Bcr-Abl-32D-EGFP+ cells (5 × 105) suspended in 200µL of HBSS were injected into each mouse through the tail vein. After 48 h of transplantation, mice were treated with vehicle (DMSO), or JMF4073 (5 mg/kg body weight) by intraperitoneal injection at 24 h interval for 14 days. Injected mice were monitored for peripheral blood (PB) and counted by Taiwan Mouse Clinic-National Phenotyping Center, National Research Program for Genomic Medicine (NSC). For T315I-Bcr-Abl CML mice, T315I-Bcr-Abl-32D-EGFP+ cells (1×106) suspended in 200 µL of HBSS were tail-vein-injected. After 7 days of transplantation, mice were treated with vehicle (DMSO), UK-5099 (10 mg/kg body weight), JMF4073 (5 mg/kg body weight), or UK-5099 combined with JMF4073 by intraperitoneal injection at 24 h interval for 14 days. For monitoring CML progress, mice blood was collected in anticoagulation tubes by submandibular blood collection, followed by lysing red blood cells with RBC lysis buffer (Invitrogen). After washing, the remaining cells were suspended in 500 [illegible]L of HBSS and subjected to flow cytometry analysis (FACScalibur, BD Bioscience) with CellQuest software. The number of EGFP+ cells were collected from 20,000 single-cell events.

Wild animals

No wild animals were used in this study.

Reporting on sex

We did not observe differences between male and female. The in vivo experiments were performed on female mice.

Field-collected samples

No field-collected samples were used in this study.

Ethics oversight

The animal studies were approved by the biosafety committee at National Taiwan University and conformed to the national guidelines and regulations (IACUC # 20201063)

Note that full information on the approval of the study protocol must also be provided in the manuscript.

Plants

Seed stocks

NA

Novel plant genotypes

NA

Authentication

NA

Flow Cytometry

Plots

Confirm that:

☐ The axis labels state the marker and fluorochrome used (e.g. CD4-FITC).

☐ The axis scales are clearly visible. Include numbers along axes only for bottom left plot of group (a 'group' is an analysis of identical markers).

☐ All plots are contour plots with outliers or pseudocolor plots.

☒ A numerical value for number of cells or percentage (with statistics) is provided.

Methodology

Sample preparation	1. Cells stained with CellROX Green for 15 min, after wash twice, cells were subjected to flow cytometry.<br>2.Cells are infected with EGFP and injected in to C3H through tail-vein. Mice blood was collected in anticoagulation tubes by submandibular blood collection, followed by lysing red blood cells with RBC lysis buffer (Invitrogen). After washing, the remaining cells were suspended in 500 ?L of HBSS and subjected to flow cytometry analysis.
Instrument	flow cytometry analysis (FACScalibur, BD Bioscience) .
Software	CellQuest software
Cell population abundance	10000 cells
Gating strategy	Cells were gated with FSC-H andSSC-A, cells were distinguished from debris. For all mice blood experiments, gating was based on two clearly distinguished population and comparison of mice without EGFP cells injection.

☐ Tick this box to confirm that a figure exemplifying the gating strategy is provided in the Supplementary Information.
